# Neuromodulation in Drug Resistant Epilepsy

**DOI:** 10.14336/AD.2021.0211

**Published:** 2021-07-01

**Authors:** Natalia Rincon, Donald Barr, Naymee Velez-Ruiz

**Affiliations:** Department of Neurology, University of Miami Miller School of Medicine, Miami, FL 33136, USA

**Keywords:** epilepsy, drug-resistant epilepsy, neuromodulation, neurostimulation, VNS, RNS, DBS

## Abstract

Epilepsy affects approximately 70 million people worldwide, and it is a significant contributor to the global burden of neurological disorders. Despite the advent of new AEDs, drug resistant-epilepsy continues to affect 30-40% of PWE. Once identified as having drug-resistant epilepsy, these patients should be referred to a comprehensive epilepsy center for evaluation to establish if they are candidates for potential curative surgeries. Unfortunately, a large proportion of patients with drug-resistant epilepsy are poor surgical candidates due to a seizure focus located in eloquent cortex, multifocal epilepsy or inability to identify the zone of ictal onset. An alternative treatment modality for these patients is neuromodulation. Here we present the evidence, indications and safety considerations for the neuromodulation therapies in vagal nerve stimulation (VNS), responsive neurostimulation (RNS), or deep brain stimulation (DBS).

## Introduction

Epilepsy affects approximately 70 million people worldwide, and it is a significant contributor to the global burden of neurological disorders [1, 2]. Epilepsy is associated with an increased risk of comorbid conditions including psychiatric disorders, cognitive disturbances, and migraine headaches, and it can have a profound impact on quality of life [3, 4]. Mortality is three times higher in people with epilepsy (PWE). Among deaths attributable to seizures, important causes include sudden unexpected death in epilepsy (SUDEP), status epilepticus, physical injuries, and drowning [5]. The risk of SUDEP is 1/1000 in PWE who have rare seizures, but in those with drug resistant epilepsy the risk increases 15 fold (18/1000) [6].

Drug-resistant epilepsy is defined by the failure of adequate trials of two tolerated, appropriately chosen and used antiepileptic drugs (AEDs) (whether as monotherapies or in combination) to achieve sustained seizure freedom [7]. Despite the availability of new AEDs, the probability of achieving seizure freedom in newly diagnosed epilepsy patients has remained unchanged over the last 30 years [8]. Approximately 50% of patients with newly diagnosed epilepsy achieve seizure freedom with the first AED, while only 11% of PWE become seizure free after the second AED, and a mere 3% stop having seizures after failing the second medication trial, leaving 30-40% of PWE with drug-resistant epilepsy [9].

Once identified as having drug-resistant epilepsy, patients should be referred to a comprehensive epilepsy center for evaluation in order to establish if they are good candidates for potential curative surgical interventions, [10, 11] or for palliative procedures expected to improve seizure control. Unfortunately, a large proportion of patients with drug-resistant epilepsy are poor candidates for resection or laser ablation due to a seizure focus located in eloquent cortex, multifocal epilepsy or inability to identify the ictal onset zone. Neuromodulation therapies are palliative nonpharmacologic options for patients who are not candidates for surgical resection or ablation. These entail electrical stimulation of specific neuroanatomical structures with the aim of affecting hyperexcitability in their circuit. Neuromodulation techniques include vagal nerve stimulation (VNS), responsive neurostimulation (RNS), and deep brain stimulation (DBS). Here we present the evidence, indications and safety considerations for these neuromodulation modalities, with an emphasis on RNS and DBS.

### Open-loop vs Closed loop

Cortical electrical stimulation has been shown to suppress epileptiform activity or reduce seizure rate following continuous electric pulses. There are two different methods of electrical delivery: open-loop and closed-loop. The open-loop approach delivers a pre-scheduled stimulation regardless of the brain electrophysiological activity [12]. The neural stimulation in DBS and VNS has been traditionally delivered through an open-loop system. Alternatively, a closed-loop system only delivers stimulation when it detects the initiation of seizure activity. RNS is delivered in a closed-loop approach. This monitoring of electrical activity is achieved through electrocorticography (ECoG) with intracranial electrodes which continuously works to identify patterns predictive of ictal activity. Cortical stimulation is only administered when ictal patterns are identified. This stimulation either prevents the seizure or stops the clinical manifestations of the ictal activity [12-14].

**Figure 1.**
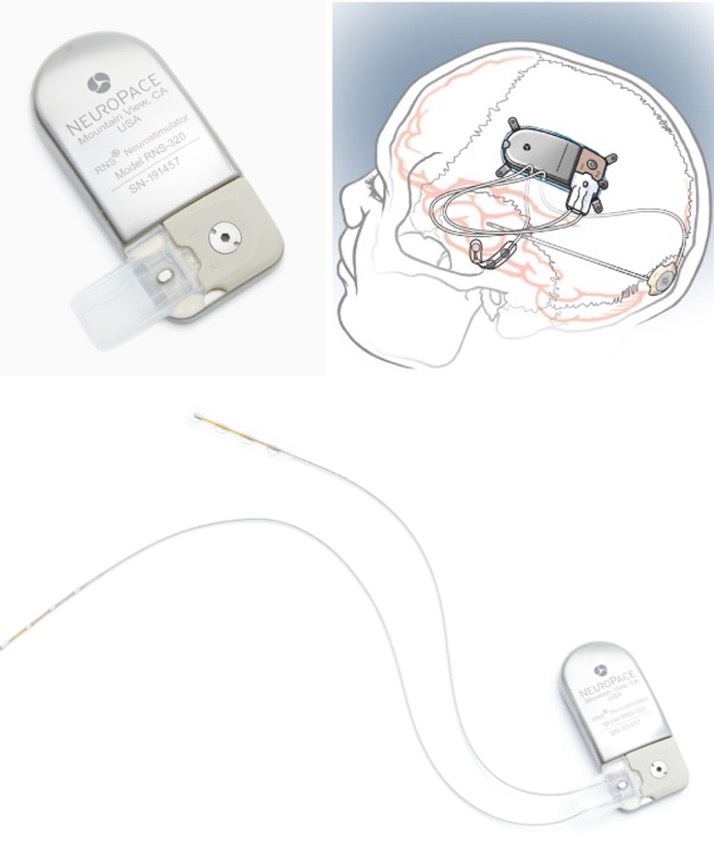
**Responsive Neurostimulator (RNS)**. The top left images show the neurostimulator; bottom image shows the neurostimulator with attached leads; top right image shows an illustration of the device placed in the skull.

## Responsive Neurostimulation (RNS)

### Background

Responsive Neurostimulation (RNS) is a closed loop system approved in 2013 for patients 18 years or older, with medically refractory seizures, and with pre-surgical work up suggesting no more than 2 epileptogenic foci [15]. The RNS system involves a stimulator connected to two depth electrodes or subdural strips that are placed intracranially at the seizure foci [16] (See Fig. 1). The procedure for placement of the RNS system also involves a craniotomy for the implantation of the neurostimulator within the skull [12, 17]. As stated above, the device continuously detects electrocorticographic activity through the electrodes and delivers programmable stimulation targeting the ictal focus to abort an impending seizure or seizure related activity [18, 19]. The settings of the device are adjusted by the physician based on the patterns of ictal onset and activity noted on the ECoG [20]. Patients also receive a remote monitor to transfer data from the neurostimulator into a secure web-based program called Patient Data Management System, where physicians are able to review it [21].

### Evidence

The efficacy of RNS was studied in three main clinical trials: an open label feasibility study, a 2-year pivotal study and a long-term trial among participants of the first two to assess for safety and efficacy. Subjects were 18-70 years old with focal onset seizures refractory to two or more AEDs, had an average of three or more disabling seizures per month, and diagnostic testing revealed 1-2 epileptogenic foci. The most common stimulation settings in the clinical trials were 100-200?Hz stimulation frequency, 1.5-3?mA current, 160?μs pulse width, and 100-200?ms burst duration. The RNS pivotal study showed a significant reduction in seizure frequency in the treatment arm compared to the sham group (37.9% vs 17.3%, p=0.012) without difference in adverse events, and seizure reduction was sustained [22]. Seizure control was also sustained through the open label period. Heck et al. found a median percent reduction in seizures of 44% at 1 year and 53% at two years [23], and Bergey et al. found a 48 to 66% reduction over years 3-6 postimplant with observed improvements in quality of life [24]. Further, treatment response was robust in mesial temporal lobe epilepsy and other brain regions with one or two seizure foci, and if patients had previous surgery or prior VNS [19, 22].

Quality of life among patients treated with RNS was also assessed as part of the pivotal study through behavioral surveys that were administered at baseline, year 1 and year 2. Patients at the end of two years reported improvements in quality of life (44%) with no significant changes in mood or suicidality. These improvements were observed regardless the area of seizure onset or antiepileptic drug use [25].

In addition to significant improvements in seizure frequency, the RNS system provides continuous monitoring of abnormal electrographic activity which may assist the clinician in seizure management. ECoG data recorded by RNS may show quantitative changes when a beneficial AED is initiated, providing an indication for a clinically meaningful reduction in seizures as early as 1-3 months [26]. These early findings suggest that data from RNS may assist clinician in optimization of AEDs. Similarly, circadian patterns of epileptiform activity using intracranial recordings captured by RNS show a strong periodicity with strong nocturnal occurrences regardless of electrode location [27]. Access to this data may assist the clinician in identifying the efficacy of a treatment and titrate medications at certain times of day [27].

### Location of Seizure Onset

#### Mesiotemporal

Patients with intractable mesial temporal lobe epilepsy who are poor surgical or ablative candidates due to bilateral seizure foci, or unacceptably high risk of memory decline, with or without hippocampal sclerosis on imaging, may benefit from RNS [16, 23]. During the pivotal study, out of the 191 subjects, 50% had seizures arising from the mesial temporal lobes with a majority of them having bilateral onset (73%) [23]. Disabling seizures were reduced by a median 66.5% at 6 years, and over the entire open-label period 45% of subjects reported seizure-free intervals lasting >3 months, and 15% were seizure free for 1 year or longer [28]. Moreover, there was no difference in seizure control between subjects with and without mesial temporal sclerosis on imaging, whether or not prior intracranial monitoring had been completed, or prior VNS treatment was pursued [28].

#### Neocortical seizures

Patients with seizures arising from eloquent cortex are at risk for neurological deficits with 17-67% of patients that undergo focal cortical resection reporting worsening or new deficits [19]. A subset of subjects from the RNS feasibility and pivotal trials with neocortical origin seizures had a median percent reduction in seizure at the end of year two of 44%, and 61-76% over 5 and 6 years respectively, with 37% of patients having a seizure free interval >=3 months during the open label period [19]. These benefits were noted in seizure foci in all neocortices (frontal and parietal 70%, temporal 58%, multi-lobar 51%), and for patients with stimulation of language areas there were no adverse events related to dysfunction of Broca’s or Wernicke’s regions. Similar to findings in mesial temporal seizures, there was no difference in patients with intracranial monitoring prior to implantation, with prior surgical resection, or VNS [19].

Notably, there was a difference in response whether or not there was a structural lesion on MRI with greater response in patients with a structural lesion (77%) compared to those without (45%). This finding is possibly due to less precise localization in patients without a structural lesion, for which further studies may elucidate if functional neuroimaging prior to implantation may help guide electrode placement and increase efficacy in these patients [19].

### Safety Considerations

RNS serious adverse events were no worse than those seen in deep brain stimulation for Parkinson’s disease, resective epilepsy surgery, or intracranial electrode implantation [21]. Adverse effects were primarily related to the nature of an implanted device or a seizure-related event. Intracranial hemorrhages were seen in 4.7% of participants, most of which occurred in the initial days after device implantation, and a small proportion of them were related to seizure related head trauma. Risk of infection per procedure has been reported to be 3.7% [ 18]. Transient adverse events related to memory were seen in 6.3% of subjects, all of which already had mild memory impairment prior to implantation of device [28]. Real-world analysis of patients with RNS for at least one year showed a comparable safety profile as that presented in the clinical trials [29].

### Candidate Selection

Neuromodulation is a palliative tool for the management of epilepsy and as a result must be considered once an extensive investigation for a potential area of resection is completed and the area is determined to be unresectable. Unresectable areas include a focus within eloquent cortex or contralateral to a previous resection, as well as an extensive brain malformation without possibility of complete resection, and an area which resection would result in unacceptable cognitive risks [16]. Patients with medically refractory seizures, with no more than two seizure foci (unilateral or bilateral) or a single focus in an eloquent, unresectable area, and frequent seizures (approximately 3 per month) are considered for RNS implantation [15, 16, 30, 31].

**Figure 2.**
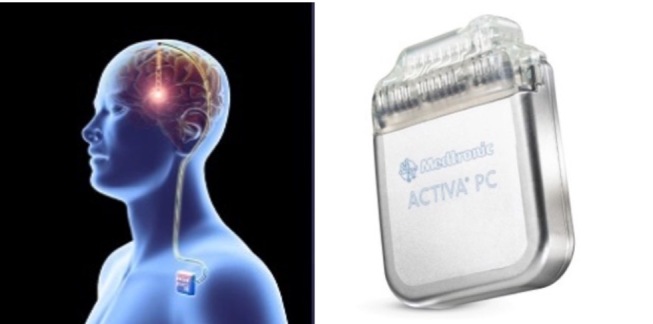
**Deep Brain Stimulator (DBS)**. The left images show the stimulator attached to the left pectoral region; the right image shows the stimulator without leads attached.

## Deep Brain Stimulation (DBS)

### Background

Deep brain stimulation is a therapeutic option approved by the FDA for use in patients with medically refractory Parkinson’s Disease and Essential Tremors in 1997 and 2002, respectively [34]. The success of this stimulatory device for the treatment of movement disorders propelled research for its use in patients with other neurological conditions including medically refractory epilepsy [34, 35]. Deep brain stimulation was approved by the FDA in 2018 for the treatment of epilepsy in patients 18 years and older based on the results of the Stimulation of the Anterior Nucleus of the Thalamus for Epilepsy (SANTE) trial which resulted in significant reduction in seizure frequency among participants during double-blind, open-label, and long-term follow up at 5 years [36, 37].

DBS functions through an open-loop system by delivering a predetermined electrical stimulation to electrodes connected to deep brain structures with a permanent generator implanted superficial to the pectoral muscle [18] (See Fig. 2). The goal is to modulate cortical excitability in an effort of reducing the frequency and severity of seizures. The selection of the structures comes from studies of cortical-subcortical networks, such as the cortical-striatal-thalamic network and the circuit of Papez, along which seizures often propagate. The notion is that these neural networks provide potential points for intervention and lesions in this circuit may interrupt the spread of seizure activity to the neocortex [39, 40].

Different targets of stimulation have been studied including the anterior nucleus of the thalamus (ANT), the centromedian nucleus of the thalamus (CMT), the hippocampus (HC), the posterior hypothalamus and the cerebellum [37, 38, 41, 42]. The usual stimulation parameters for ANT are ≥100 Hz and voltage at 1-10V; for CMNT high-frequency stimulation at voltage 1-10V; and low (10Hz) or high (200Hz) stimulation for the cerebellum [38, 41]. Though different targets remain promising, the most robust data comes from targeting the ANT and the HC [38].

### Evidence

#### Anterior Thalamic Nuclei

Deep brain stimulation was approved by the FDA in 2018 for the treatment of epilepsy in patients 18 years and older based on the Class I data of the Stimulation of the Anterior Nucleus of the Thalamus for Epilepsy (SANTE) trial. The SANTE trial was a multicenter, double blind, randomized study which enrolled patients 18-65 years old, with focal onset seizures, with a seizure frequency of at least 6 per month and no more than 10 per day, that had failed at least 3 AEDs [37]. DBS electrodes were implanted in the ANT bilaterally via stereotactic technique with a standardized implantation procedure across all centers [37].

Location of seizure onset was also varied with the majority of participants having temporal lobe seizure onset (60%); 27% frontal lobe, 4.5% parietal lobe, 9.1% diffuse or multifocal, and 9.1% other [37]. Response to treatment varied upon area of seizure onset. Among patients with seizure origin in one or both temporal regions the median seizure reduction in the stimulated group was 44.2% compared to 21.8% in the control group. For patients with diffuse or multifocal seizure origin there was a 35% reduction in the intervention group compared to controls (14.1%). There was no significant difference in reduction in seizure frequency between patients with frontal, parietal or occipital regions. During the long-term follow up period there was a median change in seizure frequency of 41% from baseline at 13 months and 56% at 25 months with a proportion of participants achieving seizure freedom for a 6-month period (13%), at least a year (7.3%), at least 2 years (3.6%) and one patient for more than 4 years (0.9%). [37]

Out of all participants enrolled in SANTE, 44.5% had a previous VNS implant and 24.5% had a previous epilepsy surgery [37]. There was no difference in response among patients with prior history of VNS and/or resective epilepsy surgery and those without. There was no distinct association between response to DBS and underlying AED treatment though the study was not powered for this [34].

#### Hippocampus

Similar to the ANT, the hippocampus is a continuous focus of research in the propagation of seizure activity due to its role in the Papez circuit and the potential for its electrical stimulation halting the progression of a seizure event originating from the mesial temporal region, a highly epileptogenic focus [38, 40, 43]. Smaller studies have addressed its benefit among patients with mesial temporal lobe epilepsy, the most common form of medically refractory epilepsy [44-46]

Tellez-Centeno et al identified a median seizure reduction of 15% among a group of 4 participants with medically intractable MTLE related to mesial temporal sclerosis with contraindications to resective surgery that underwent electrical stimulation of the hippocampus. These results however were not statistically significant, though possibly limited by the small sample size [44]. Boon et al followed 12 patients with MTLE for a mean follow up period of 31 months where 6 patients had a seizure reduction >=50-90%, one achieved seizure freedom for >1 year and two had a 30-49% reduction; one was a non-responder. There were minimal adverse events with the exception of asymptomatic intracranial hemorrhage in one patient [46]. Velasco et al followed 9 patients with focal seizures with and without hippocampal sclerosis on MRI that underwent stimulation of the hippocampus. Though sample size was small, they found improvement in both types of patients, where those with hippocampal sclerosis had residual seizures and those without achieved seizure freedom [45]. A larger double-blind RCT by Cukiert followed 16 patients with refractory MTLE for a longer blinded period (6 months) where 50% of those in the intervention arm became seizure free. In contrast to Velasco et al this study showed better response among patients with hippocampal sclerosis [47]. While the current evidence suggest that hippocampal stimulation is effective at reducing seizure frequency among patients with refractory MTLE that are poor surgical candidates, larger randomized controlled trials are needed to better characterize the benefits of this approach among patients.

#### Centromedian Nucleus of the Thalamus

Centromedian Nucleus of the Thalamus has been investigated as a potential therapeutic target given its widespread projection to the cortex, insula and basal ganglia [40]. The current data from smaller studies supports the use of CMT-DBS among patients with generalized epilepsy, and Lennox-Gastaut Syndrome (LGS). The most notable study comes from Velasco et al. who reported the first cases of CMT-DBS in 1987, with subsequent series supporting the initial results. They found significant seizure reduction among patients with generalized tonic-clonic seizures, but no change was noted in patients with focal seizures [48]. A subsequent study in 13 patients showed an overall 80% reduction in seizure frequency with this approach [42]. Valentin et. al described a decrease in seizure frequency of >50% among patients with generalized epilepsy during both blind and open-label periods. These findings supported those of Velasco regarding the apparent benefit of CMT-DBS among patients with generalized seizures refractory to treatment.

#### Subthalamic nucleus/Substantia Nigra

The subthalamic nucleus (STN) has been studied for the treatment of Parkinson’s disease and obsessive-compulsive disorder and its efficacy in the treatment of intractable epilepsy remains unclear. Animal models have shown involvement of the STN in the development of motor seizures suggesting its possible utility for stimulation in patients with refractory epilepsy [49]. Case reports have shown varying degrees of positive results [50-52]. Benabid et al. first reported a case of a 5-year-old girl with intractable epilepsy due to a centroparietal dysplasia who experienced an overall 80% reduction in seizure frequency following high frequency stimulation of the STN [50]. Similarly, Chabardès et al. followed 5 patients of which 3 responded with a 67-80% reduction in seizure frequency, including one with severe myoclonic epilepsy though at a lower rate [53]. Vesper et al. (2007) reported a 50% reduction in seizure frequency in a patient with progressive myoclonic epilepsy (PME) with refractory seizures who underwent implantation of bilateral DBS electrodes in the STN [52]. Wille et al. followed 5 adult patients with PME for a median 24 months and reported a reduction in myoclonic seizures between 30-100%. Though results for the use of STN-DBS are promising, especially among patients with myoclonic epilepsy, larger studies are needed to elucidate the utility of this approach.

### Safety considerations

The most common adverse events related to DBS-ANT in SANTE included surgical related complications such as implant site pain (10.9%), implant site infection (12.7%), and incidentally found intracranial hemorrhages (4.5%) on imaging without clinical findings [24]. The most common device related side effects reported by participants in the initial trial and long term follow up were paresthesias (18.2%), subjective memory impairment, and depressed mood [36, 37]. A larger proportion of patients in the intervention group had memory complaints; however subsequent follow up studies revealed no objective evidence of neurocognitive decline or depression scores among these patients [54]. Other complications include lead fracture, lead side fibrosis, electrode migration, external interference with other devices, transient worsening or new seizures and dizziness [41]. The rate of these adverse events is similar to that seen in patients undergoing DBS for movement disorders [41]. Similar adverse event profile was noted among patients that received HC and CMT stimulation [38, 55].

### Candidate selection

In an effort to modulate cortical excitability and reduce frequency and severity of seizures, multiple targets have been studies in the past decades for the treatment of intractable epilepsy. However, DBS of the anterior thalamic nucleus has the most robust evidence to date and is currently the only DBS approach approved by the FDA. It is offered to patients 18-65 years old with drug resistant epilepsy who are experiencing at least 6 seizures per month and no more than ten per day, with impaired quality of life over 12-18 months. Patients with focal epilepsy with >2 identified or nonlocalizable epileptogenic foci are preferred candidates for DBS. The targeting of particular locations based on epilepsy syndrome has so far come from smaller studies and further exploration is required. Based on the current data, future studies are needed to expand on the findings pointing at the benefit from specific target based on epilepsy syndrome including CMT stimulation for patients with generalized epilepsy and Lennox-Gastaut, and SNT stimulation in patients with myoclonic epilepsy [45, 47, 51, 52, 55].

## Vagus Nerve Stimulation (VNS)

### Background

**Figure 3.**
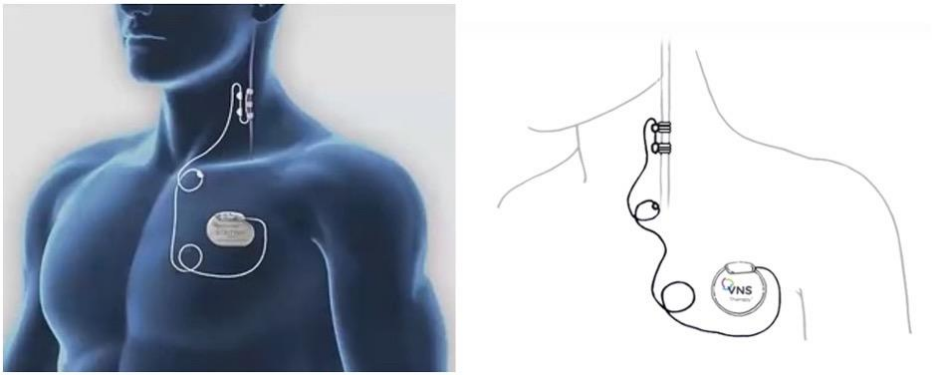
**Vagus Nerve Stimulator (VNS)**. Stimulator implanted subcutaneously in the left anterior chest wall with stimulating electrodes coiled around the left vagus nerve.

Vagus Nerve Stimulation (VNS) was the first implantable device for the treatment of epilepsy in patients that have failed two or more AEDs without adequate control and that are poor surgical candidates. It was approved in 1997 for patients >12 years of age and was expanded to include patients >4 years of age as of 2017. VNS consists of stimulating electrodes that are coiled around the left vagus nerve in the carotid sheath and implanted subcutaneously in the left anterior chest wall [18, 56, 57](See Fig. 3). The left vagus nerve is selected due to right vagus nerve involvement with sinoatrial node innervation and attempt at limiting the risk of bradycardia and arrythmias [18, 58]. Stimulation is delivered in an open-loop approach with scheduled stimulation to the vagus nerve administered every few minutes. Stimulation is typically started at 0.5 mA and increased to 1.25-2.0 mA over several weeks [58]. Patients are provided a magnet that may be used to provide an additional stimulation if patient experiences an aura or by a companion if patient has a witnessed seizure [59]. Newer generations VNS devices have added new features including the administration of additional stimulations in response to increases in heart rate given the association between ictal periods and tachycardia [18]. Lastly, since the approval of VNS for the treatment of epilepsy, the FDA has given approval of this device for the treatment of depression and more recently of cluster headaches [60].

### Evidence

The evidence around VNS can be divided into short and long-term outcomes. Since 1994 multiple studies have assessed the efficacy and safety of VNS. The largest studies, E03 (multinational) and E05 (US Centers) were of similar design and followed response in patients with refractory epilepsy, which was described as at least 6 focal onset seizures involving loss of awareness in a 30-day period with no more than 21 days in between [56, 59, 61-64]. They were observed for a 3-month baseline period followed by 3 months post-implantation where participants received high (average 1.3 mA) versus low stimulation (average 1.2mA, less stimulation and pulse frequency) [63]. Results from these studies showed a mean seizure reduction of 24.5% to 27.90% in the active group compared to controls (6.1% to 15.2%), for E03 and E05 respectively [59, 62, 63]. For E05, 31% of patients in the high frequency group experienced a >50% reduction in seizure frequency compared to 13% in the low frequency group (p=0.02), and for E03, 10.6% of patients in high-frequency group achieved a statistically significant >=75% reduction compared to low frequency group (2.0%); decrease of >=50% in seizure reduction was not statistically significant [62, 63].

Long term studies of the initial trials demonstrated increase in median seizure reduction over time [39, 65-67]. Kawai et al described a median seizure reduction of 25%, 40.9%, 60% and 66.2% at three, six, twelve, twenty-four and thirty-six months of VNS therapy [68]. Similarly, a large-scale evaluation of the VNS Patient Outcome Registry showed a progressive improvement in responsiveness (described as =>50% seizure reduction) where at 0-4 months post-implant, 49% responded to VNS with 5.1% becoming seizure free, and at 24-48 months, 63% responded with 8.2% achieving seizure freedom. These findings were most remarkable among patients with age onset >12 years old (OR 1.89, 95% CI 1.38-2.58) predominantly generalized seizure type (OR 1.36, 95% CI 1.01-1.82), and non-lesional (OR 1.38, 95% CI 1.06-1.81) [69]. Studies have also shown benefit of VNS in epilepsy syndromes including Lennox-Gastaut and genetic generalized epilepsy syndromes [70-73].

### Safety Considerations

VNS tends to be well tolerated among patients. Adverse reactions related to device implantation in the acute setting include infection, vocal cord paresis, and lower facial weakness [58, 64, 74]. More commonly reported side effects include hoarseness, cough, voice alteration and throat pain [63, 64]. Case series have shown an increase in apnea-hypopnea index with activation of VNS causing sleep apnea or exacerbating an underlying diagnosis [75], and cardiac arrythmias have been reported resulting in decreased levels of stimulation [60]. Most VNS side effects are related to stimulation level with overall trend to decrease overtime with rare necessity for device explanation [58].

Baseline electrocardiogram and in some cases sleep studies are obtained as VNS may change respiratory patterns, increasing the number of apneic hypopneic events, though presently only severe sleep apnea is considered a contraindication[39, 60, 75] Relative contraindication to VNS implantation exists among patients with left vagus nerve injury, and left vagotomy is an absolute contraindication[18, 60] Other exclusion criteria includes severe heart disease including bradyarrhythmia, history of dysautonomia, asthma or severe sleep apnea. [33]

### Patient Selection

VNS is approved for patients (4 or older in the United States) with medically refractory focal and generalized seizures who are poor surgical candidates for resective or ablative surgeries. As localization of seizure focus is not required for this adjunctive treatment option, VNS should be considered in patients with unlocalizable or multifocal epilepsy [33]. While most patients in clinical trials treated with VNS have had seizures of focal onset, evidence suggests that patients with non-lesional epilepsy, primary generalized epilepsy and Lennox-Gastaut Syndrome may be suitable candidates [60, 71, 72]. Also, consideration should be given to VNS among potential candidates with comorbid depression (Table 1).

**Table 1:** Comparison between different features of VNS, RNS and DBS.

	VNS	RNS	DBS
**Indication**	Focal and generalized; unlocalizable or multifocal	Focal; up to 2 seizure foci (unilateral or bilateral) or a single focus in an eloquent, unresectable area.	Focal and generalized; >=2 identified epileptogenic foci
**Loop Types**	Open	Closed	Open
**Target**	Left anterior chest wall; coiled around left vagus nerve	Two depth electrodes or subdural placed intracranially at the seizure foci	Anterior Nucleus of the Thalamus (ANT)
**Stimulation Parameters**	Start at 0.5 mA and increase to 1.25-2.0 mA over weeks; may provide additional stimulation with magnet	100-200?Hz stimulation frequency, 1.5-3?mA current, 160?μs pulse width, and 100-200?ms burst duration	ANT simulation: frequency ≥100 Hz and voltage at 1-10 V
**Side Effects/Complications**	Hoarseness, cough, voice alteration and throat pain; exacerbation of OSA, cardiac arrythmias.	Infection, post-device implantation ICH, transient memory impairment	Paresthesia, subjective memory impairment, and depressed mood; implant site pain and infection, incidentally found ICH
**MRI compatible**	Yes	No (newer devices compatible)	Yes

## Discussion

Epilepsy is one of the most common neurological disorders of the brain. It is associated with an increased risk of mortality and psychiatric, cognitive and psychosocial comorbidities. Despite the advent of new AEDs, drug resistant-epilepsy continues to affect 30-40% of PWE [9]. Once identified as having drug-resistant epilepsy, these patients should be referred to a comprehensive epilepsy center for evaluation to establish if they are candidates for potential curative surgeries [10, 11]. Unfortunately, a large proportion of patients with drug-resistant epilepsy are poor surgical candidates due to a seizure focus located in eloquent cortex, multifocal epilepsy or inability to identify the zone of ictal onset. An alternative treatment modality for these patients is neuromodulation. Neuromodulation modalities include RNS which may be considered in patients with up to two well-localized epileptogenic foci or single focus in eloquent area; DBS which can be selected in patients with limbic epilepsy with poorly localized epileptogenic areas or more than two epileptogenic foci; and VNS which is an option for patients who refuse to be considered for the above options, or in those with more than two foci, generalized epilepsy, and Lennox-Gastaut syndrome. At this point in time, more experience is required to determine the optimal choice of neuromodulatory treatment in patients with drug resistant epilepsy that are poor surgical candidates. Future study directions should include head-to-head trials comparing these three different modalities of neuromodulation.
